# Integrated modelling of age and sex patterns of European migration

**DOI:** 10.1111/rssa.12177

**Published:** 2016-01-22

**Authors:** Arkadiusz Wiśniowski, Jonathan J. Forster, Peter W. F. Smith, Jakub Bijak, James Raymer

**Affiliations:** ^1^ University of ManchesterUK; ^2^ University of SouthamptonUK; ^3^ Australian National UniversityCanberraAustralia

**Keywords:** Bayesian modelling, Contingency tables, Europe, International migration statistics, Migration models

## Abstract

Age and sex patterns of migration are essential for understanding drivers of population change and heterogeneity of migrant groups. We develop a hierarchical Bayesian model to estimate such patterns for international migration in the European Union and European Free Trade Association from 2002 to 2008, which was a period of time when the number of members expanded from 19 to 31 countries. Our model corrects for the inadequacies and inconsistencies in the available data and estimates the missing patterns. The posterior distributions of the age and sex profiles are then combined with a matrix of origin–destination flows, resulting in a synthetic database with measures of uncertainty for migration flows and other model parameters.

## 1 Introduction

Age and sex patterns of migration are important for understanding the types and motives of migrants. These patterns are also required for population planning and for designing policies to attract or restrict migration. The current state of migration data, however, prevents comparative analyses across countries, as each country essentially collects its own data to suit its own purposes. This results in migration statistics being produced by different mechanisms of data collection (e.g. administrative registers, surveys and censuses) and criteria to qualify migrants. Migration data have a long history of being problematic and inconsistent (United Nations, 1949; Kelly, 1987).

With the formation and recent expansion of the European Union (EU), there has been renewed interest in overcoming the inconsistencies in measurement (Poulain *et al*., 2006; Kupiszewska and Nowok, 2008; Kupiszewska and Wiśniowski, 2009) and developing models for estimating missing flows (Raymer, 2008; Cohen *et al*., 2008; Abel, 2010; De Beer *et al*., 2010; Raymer *et al*., 2011, 2013). This has been bolstered by Regulation 862/2007 of the European Parliament and of the Council of July 11th, 2007, on the provision of migration statistics, which went into effect for 2009 reported figures.

In this paper, we respond to the need for better and more detailed migration data by developing a Bayesian hierarchical Poisson model with overdispersion to estimate the age and sex distributions of international migration in the EU and European Free Trade Association (EFTA). This work utilizes the results of another model that was previously developed to estimate the overall levels and spatial patterns of migration in the EU and EFTA from 2002 to 2008 (Raymer *et al*., 2013). Estimating the age and sex patterns of migration represents the logical next step as these structures are essential for understanding the drivers of population change and the heterogeneity of the migrant groups. As with the measurement of the levels and spatial patterns, the age and sex distributions of migration also suffer from inconsistencies in measurement and missing data.

The organization of this paper is as follows. In Section 2, we provide a background on modelling and estimating the age and sex profiles. Section 3 is a description of the data underlying our estimates. In Section 4, we present the modelling framework that was utilized to distribute the origin–destination flows by age and sex. Section 5 contains results with measures of model assessment. In the last section we conclude and suggest directions for future research.

The data that are analysed in the paper and the programs that were used to analyse them can be obtained from

http://wileyonlinelibrary.com/journal/rss-datasets

## 2 Background

Europe is a diverse and unique area of the world. Countries in eastern Europe are facing population decline from very low and sustained fertility levels and net emigration, whereas many populations in western Europe are growing because of its attractiveness and opportunities to migrants. Europe also contains the EU and the EFTA with 31 member countries having the right of free movement and residence within the system. Around 3 million–6 million people each year (interquartile range) are estimated to migrate to these countries from other member countries and from across the world (Raymer *et al*., 2013).

As a world region, Europe is ideal for studying migration because it has a relative abundance of migration data for a large group of countries close together. However, even here, reported statistics on migration can be confusing or even non‐existent. This is caused by the absence of a consensus on how migration should be measured. As a result, comparative analyses are hindered by differing national views concerning the definition of a migrant. Furthermore, migration data are collected by using a variety of sources, including administrative registers, censuses or surveys. A compelling approach for diagnosing conflicts between sources of data in a hierarchical framework was introduced by Presanis *et al*. (2013). We are interested in migration data, where the conflicts are known (see, for example, Kelly (1987)) and hence our focus is on constructing a model to accommodate such conflicts adequately.

To overcome the problems of inconsistent migration data, there are two possible solutions. First, national statistical offices in different countries could communicate with each other like they do in the Nordic population registers, where Denmark, Finland, Iceland, Norway andSweden all exchange information on their international migrants by notifying the sending country when someone has registered in their system. Hence, at least in principle, a person should be included only on one Nordic population register at a time. All other national statistical offices in the world rely on their own independent systems and measurements to track migration flows from and to other countries, resulting in inconsistencies and inaccuracies in the migration statistics.

The second option is to use models to reconcile the different reported figures and to estimate the missing data. For European migration, first attempts at bringing these two aspects together can be found in Raymer (2008), Abel (2010), De Beer *et al*. (2010) and Raymer *et al*. (2011). These works set the foundation for the integrated model of European migration for estimating origin–destination flows with measures of uncertainty (Raymer *et al*., 2013). In this model, a set of unobserved true flows of migration was estimated on the basis of four pieces of information: flows reported by the sending country, flows reported by the receiving country, covariate information and expert judgements. The reported data were harmonized via two measurement models: one for sending country data and one for receiving country data. These models took into account definitions of duration that are used in various countries, the relative accuracy of the data collection mechanisms, the overall undercount of migration and the coverage of migration. Expert judgements were also obtained and used to inform the measurement model (Wiśniowski *et al*., 2013).

In terms of measurement, the integrated model of European migration produced harmonized flows which were consistent with the United Nations (1998), page 18, recommendation for long‐term international migration, i.e. a long‐term migrant is

‘a person who moves to a country other than that of his or her usual residence for a period of at least a year (12 months), so that the country of destination effectively becomes his or her new country of usual residence’.

Finally, a migration model based on theory was used to augment the measurement model and to estimate the missing flows.

In this paper, we estimate migration flow tables with age and sex characteristics. Age patterns of migration may be interpreted within a life course framework in which individuals pass through different states of existence between birth and death (Courgeau, 1985; Willekens, 1999). This includes leaving the parental home and various (and mostly young adult) life events tied to education, employment, marriage and family formation or devolution, as well as entry into retirement and care facilities towards the later stages of life. As these transitions are fairly common across population, age‐specific patterns of migration are remarkably persistent (Rogers and Castro, 1981). For example, migrations due to marriage and education are concentrated between the ages of 18 and 30 years and are essentially unimodal in age profile. Migrations caused by changes in employment are often accompanied by spouses and children. Finally, migrations related to retirement and health are concentrated in older years of life (ages 60 years and older).

Sex distributions of migration are influenced by the types of migration, measured either from the point of view of the individual migrant (migration motives) or from that of the receiving country (admission categories). Furthermore, migration policies and attitudes towards gender roles affect male and female migrants differently. Despite the quite persistent idea that migration is dominated by young adult men, nowadays, women make up only slightly less than half of all migrants world wide, as measured by population stock data by country of birth (International Organization for Migration, 2008). Reported Eurostat migration flow data show that men have a slight majority, although there are considerable variations in individual flows.

## 3 Data

Migration flows by origin, destination, age (in 18 5‐year groups) and sex were obtained from the Eurostat database for all EU and EFTA countries that provided data between 2002 and 2008. This amounted to around 50% of the countries being covered with recent years (e.g. 2007 and 2008) containing more data than earlier years (e.g. 2002 and 2003). To maintain the aggregate levels of reported migration, the relatively small amount of flows with unknown origins or destinations were proportioned across observed patterns.

**Figure 1:**
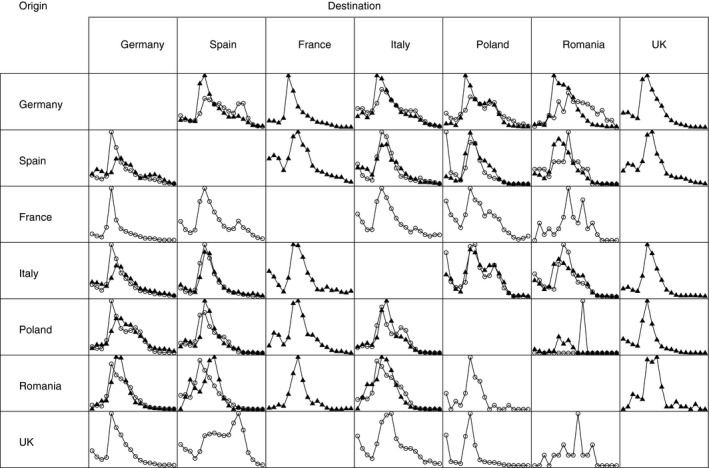
Proportions of migrations of females in each age group among the seven largest countries in the EU and EFTA, 2008: reports by sending country (▵) and receiving country (◯)

In Fig. 1, the proportion of females in each age group is presented for migration between the seven largest countries in the EU and EFTA in 2008. We find large discrepancies between age schedules that are reported by sending and receiving countries. In particular, large differences are observed for flows between Poland and Germany (the age profile for Poland is flatter than that reported by Germany), from Germany to Spain (German data do not exhibit a large proportion of migration in retirement ages) and all flows that are associated with Romania. For some flows there is only one reported schedule (e.g. from Germany to France), whereas in other cases there is no information (e.g. from France to the UK).

For the same set of flows as presented in Fig. 1, available percentages of migration of females are shown in Table 1 by sending, *E*, and receiving, *I*, country reports. Here also, we observe substantial discrepancies in the reported figures and in the patterns of missing data. For example, females are more prevalent in the receiving country report for migration from Spain to Italy than in the sending country report. For migration from Spain to Poland, the opposite pattern is found. A null percentage for flows from Romania to Poland results from no female migrants reported by Romania (only one male was reported in total).

**Table 1:** Percentage of female migration among the seven largest countries in the EU and EFTA, 2008: reports by sending country, *E*, and receiving country, *I*

*Country of origin*	*Results (%) for the following destination countries*:													
	*Germany*		*Spain*		*France*		*Italy*		*Poland*		*Romania*		*UK*	
	*E*	*I*	*E*	*I*	*E*	*I*	*E*	*I*	*E*	*I*	*E*	*I*	*E*	*I*
Germany			49	50	49		41	48	30	32	35	42	47	
Spain	48	46			50		48	54	63	28	59	34	47	
France		47		52				52		31		27		
Italy	45	40	43	58	50				51	37	47	41	46	
Poland	45	34	30	51	36		51	69			53	13	23	
Romania	78	38	61	51	65		69	54	0	42			72	
UK		44		51				46		24		42		

**Figure 2:**
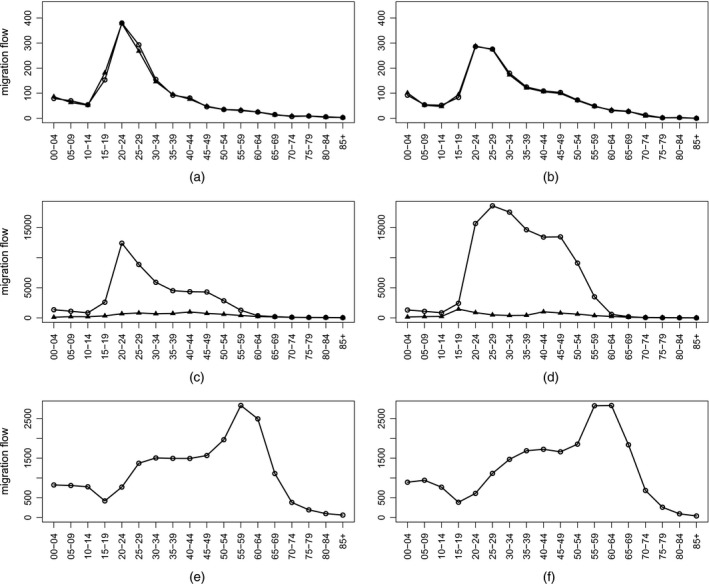
Age–sex profiles of reported migration from (a), (b) Finland to Sweden, (c), (d) Poland to Germany and (e), (f) the UK to Spain, 2006—reports by sending country (▵) and receiving country (◯): (a), (c), (e) females; (b), (d), (f) males

In Fig. 2, the reported migration flows by age and sex from Finland to Sweden, Poland to Germany and the UK to Spain are presented for the year 2006. The data from both origin and destination countries match each other almost perfectly for the Finland to Sweden flow. This is due to the exchange of information on migration flows between these countries and consistency in the definitions that are used to qualify migrants. For the age–sex profiles of migration from Poland to Germany, we observe large discrepancies between the two country reports. They result from the very different definitions of migration that are used in both countries, i.e. ‘no time limit’ specified in Germany, and ‘permanent’ migration in Poland. Finally, only flows reported by Spain are shown, as the UK did not provide age characteristics of origin–destination flows because of the relatively small sample size of its International Passenger Survey.

## 4 Methodology

**Figure 3:**
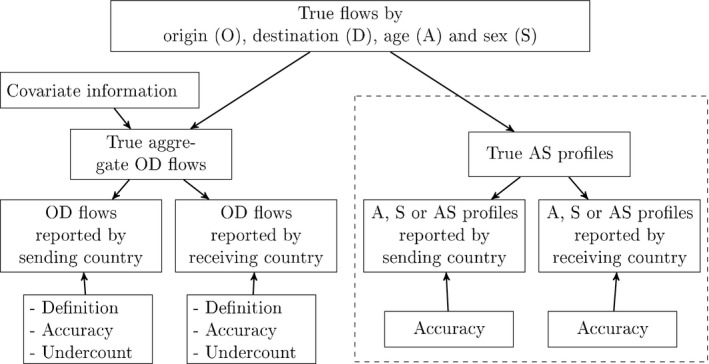
Conceptual framework for modelling migration flows

The objective of the model that is developed in this paper is to add age *A* and sex *S* categories to existing migration flow tables by origin and destination, OD, over time *T*, resulting in a five‐dimensional table of flows denoted by ODAST. The conceptual framework of the ODAS model (without time) is presented in Fig. 3. Here, the OD‐model (the left‐hand side of Fig. 3) integrates the data on migration flows reported by sending and receiving countries, covariateinformation for missing flows, and elicited expert opinion on definitions, accuracy and undercount (Wiśniowski *et al*., 2013). Definitions include duration‐of‐stay criteria and coverage. Accuracy in the OD‐model comes from both data and expert judgements. Here, on the basis of information contained in detailed reviews by Poulain *et al*. (2006) and Kupiszewska and Nowok (2008), registers were considered more precise in some countries than in others and in comparison with surveys. Undercount relates to the underregistration of immigrants within the EU, as well as the more severe problem of lack of deregistration of emigrants. The AS|OD model (the right‐hand side of Fig. 3), which is the focus of this paper, integrates the age, sex or age–sex patterns of migration into true flows. In this model, accuracy comes from the data, i.e. from the differences that are found in the sending country and receiving country reports.

Our ODAS model is factorized into a model for marginal spatial patterns (origin–destination, OD) and a model for the flows disaggregated by age and sex (AS|OD). The advantages are as follows. The modelling of the OD‐tables can be aided by the existing theories such as gravity models (Cohen *et al*., 2008) and is more amenable to the elicitation of expert opinion. Similarly, modelling of the age and sex profiles can rely on well‐documented empirical regularities ofmigration (Rogers and Castro, 1981). It is also computationally simpler to estimate the AS|OD model than the full ODAS model because of the high level of missing observations and inconsistencies in the ODAS data. Both factors, OD and AS|OD, can be combined without loss of accuracy in the estimates.

### 4.1 Statistical modelling framework

In this section, we specify the model for estimating age and sex patterns and its linkage with the origin–destination model that was described by Raymer *et al*. (2013). The study encompasses flows between 31 European countries and flows to and from the rest of the world for 18 age groups and sex between 2002 and 2008.

The data of interest can be conveniently expressed in a multi‐dimensional contingency table showing the origin‐to‐destination by age–sex flows with the cell counts corresponding to the number of migrants in a specified period. We observe counts (flows) $z_{odast}^{k}$ from country *o* to country *d* of sex *s* and in age group *a* during year *t* reported by either the sending *S* or receiving *R* country, where *k* ∈ {*S*,*R*}.

Some countries report only the sex distributions (but not age), i.e. $z_{od+st}^{k}$, where ‘+’ denotes summation over a given index. The reverse situation, when a breakdown by age is available but not by sex, occurs only for Portugal in 2008, a case that we omit. The data are not used twice, i.e. we use $z_{od+st}^{k}$ only in the absence of $z_{odast}^{k}$.

**Figure 4:**
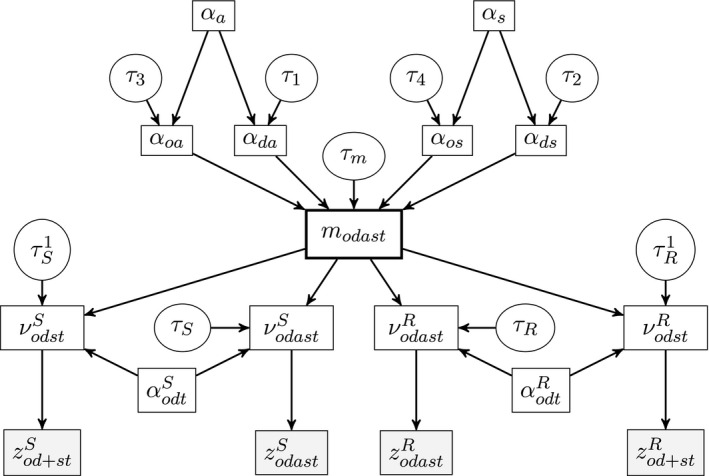
Conceptual framework for modelling age–sex profiles

For $z_{odast}^{k}$, we assume that

(1) $$z_{odast}^{S}∼\text{multinomial}\left(z_{od++t}^{S},\rho_{odt}^{S}\right),$$

(2) $$z_{odast}^{R}∼\text{multinomial}\left(z_{od++t}^{R},\rho_{odt}^{R}\right),$$

where vectors $\rho_{odt}^{k}=\left(\rho_{od1Ft}^{k},\ldots ,\rho_{odAMt}^{k}\right)$ are age–sex distributions for either the sending or receiving country, *M* and *F* in the subscript denote males and females respectively, and *A* is the oldest age group. The elements of each of the vectors sum to 1. Following Forster (2010), we respecify this model as a Poisson model. The modelling framework of the respecified AS‐model is presented in Fig. 4. We therefore assume that $z_{odast}^{k}$ follows a Poisson distribution

(3) $$z_{odast}^{S}∼\text{Poisson}\left(\nu_{odast}^{S}\right),$$

(4) $$z_{odast}^{R}∼\text{Poisson}\left(\nu_{odast}^{R}\right),$$

where

(5) $$\rho_{odast}^{k}=\nu_{odast}^{k}/\nu_{od++t}^{k}$$

and $\left\{\nu_{od++t}^{k}\right\}$ are *a priori* independent of $\left\{\rho_{odast}^{k}\right\}$ which is achieved by incorporating the three‐factor ODT‐interaction in a log‐linear model for $\left\{\nu_{odast}^{k}\right\}$, with a prior which is approximately uniform.

Let $m_{odast}$ be a true flow of migration in age‐sex group *as* from country *o* to country *d* in year *t*. It includes migration flows to and from the rest of the world (category *o*=0). In general, we are interested in obtaining the true age and sex distribution of the given origin and destination flow in year *t*, i.e. a vector $\pi_{odt}=\left(\pi_{od1Ft},\ldots ,\pi_{odAMt}\right)$, whose elements sum to 1. This can be then computed as

(6) $$\pi_{odast}=m_{odast}/m_{od++t}.$$

We assume that the Poisson mean $\nu_{odast}^{k}$ is related to the true flow $m_{odast}$ through the log‐linear model when the data by age and sex are observed:

(7) $$\text{log}\left(\nu_{odast}^{S}\right)∼N\left\{\text{log}\left(m_{odast}\right)+\alpha_{odt}^{S},\tau_{S}\right\}\;,$$

(8) $$\text{log}\left(\nu_{odast}^{R}\right)∼N\left\{\text{log}\left(m_{odast}\right)+\alpha_{odt}^{R},\tau_{R}\right\}\;,$$

where *τ* denotes precision, i.e. inverse variance. This log‐normal specification introduces overdispersion to reflect better the variability of the data. The magnitude of the overdispersion isassumed to be different for sending, $\tau_{S}$, and receiving, $\tau_{R}$, countries. Then, from expressions(5), (7) and (8) it follows that, for any multivariate logit transformation, we have

(9) $$E\left\{\text{logit}\left(\rho_{odast}^{S}\right)\right\}=E\left\{\text{logit}\left(\rho_{odast}^{R}\right)\right\}=\text{logit}\left(\pi_{odast}\right).$$

Parameter $\alpha_{odt}^{k}$ is the required three‐factor interaction to ensure a valid multinomial model. For the migration to and from the rest of the world (labelled as ‘country 0’) there is only one equation per outflow and inflow respectively, i.e.

(10) $$\text{log}\left(\nu_{o0ast}^{S}\right)∼N\left\{\text{log}\left(m_{o0ast}\right)+\alpha_{o0t}^{S},\tau_{S}\right\}\;,$$

(11) $$\text{log}\left(\nu_{0dast}^{R}\right)∼N\left\{\text{log}\left(m_{0dast}\right)+\alpha_{0dt}^{R},\tau_{R}\right\}\;.$$

The age–sex patterns of the OD true flows of migration may be modelled by using a multiplicative model, additive on the logarithmic scale. Here, the model is

(12) $$\text{log}\left(m_{odast}\right)∼N\left(\alpha_{da}+\alpha_{ds}+\alpha_{oa}+\alpha_{os},\tau_{m}\right)\;.$$

The prior distributions for parameters $\alpha_{da}$, $\alpha_{ds}$, $\alpha_{oa}$ and $\alpha_{os}$ are specified as follows:

(13) $$\alpha_{da}∼N\left(\alpha_{a},\tau_{1}\right),$$

(14) $$\alpha_{ds}∼N\left(\alpha_{s},\tau_{2}\right),$$

(15) $$\alpha_{oa}∼N\left(\alpha_{a},\tau_{3}\right),$$

(16) $$\alpha_{os}∼N\left(\alpha_{s},\tau_{4}\right),$$

where $\alpha_{a}$ and $\alpha_{s}$ are parameters capturing the age and sex patterns across all countries, which are then adjusted for a given origin and destination by the interaction parameters. This hierarchical specification allows borrowing of strength between countries to estimate the missing data patterns.

The *a posteriori* level of the true flows by origin, destination, age, sex and time can then be computed as

(17) $$y_{odast}=\pi_{odast}y_{odt}=\frac{m_{odast}}{m_{od++t}}y_{odt},$$

where the marginal flows $y_{odt}$ are the posterior samples of the true flows by origin, destination and time from the OD‐model, which harmonizes the flows and benchmarks them to the United Nations (1998) definition.

### 4.2 Including data disaggregated by sex

For some countries, the origin–destination data are available disaggregated only by sex but not by age and sex, i.e. $z_{od+st}^{k}$. We assume that these data follow a Poisson distribution:

(18) $$z_{od+st}^{S}∼\text{Poisson}\left(\nu_{odst}^{S}\right),$$

(19) $$z_{od+st}^{R}∼\text{Poisson}\left(\nu_{odst}^{R}\right),$$

where $\nu_{odst}^{S}$ and $\nu_{odst}^{R}$ are means that are not age specific. The logarithms of these sex‐specific means are assumed to be normally distributed:

(20) $$\text{log}\left(\nu_{odst}^{S}\right)∼N\left\{\text{log}\;\;\left(\sum_{a=1}^{A}m_{odast}\right)+\alpha_{odt}^{S},\tau_{S}^{1}\right\}\;\;,$$

(21) $$\text{log}\left(\nu_{odst}^{R}\right)∼N\left\{\text{log}\;\;\left(\sum_{a=1}^{A}m_{odast}\right)+\alpha_{odt}^{R},\tau_{R}^{1}\right\}\;\;.$$

We also assume that precisions $\tau_{S}^{1}$ and $\tau_{R}^{1}$ are different from those for the data with age–sex profiles available. This approach can be interpreted as a situation in which the accuracy of $z_{od+st}^{k}$ differs from $z_{odast}^{k}$. The $z_{od+st}^{k}$‐counts are disaggregated by age on the level of the true flows, i.e. by summing $m_{odast}$ by age in distributions (20) and (21), rather than aggregating the Poisson means separately for the sending and receiving country data. In other words, the missing age profiles are estimated from the age and sex‐specific data reported by other countries.

For $\alpha_{a}$ and $\alpha_{s}$ we assume standard normal priors *N*(0,1), except for the parameter $\alpha_{F}$ for females and $\alpha_{1}$ for the youngest age group, which are constrained to 0 to ensure identifiability. For the precision parameters $\tau_{m}$, $\tau_{S}$, $\tau_{R}$, $\tau_{S}^{1}$ and $\tau_{R}^{1}$ we assume an approximately non‐informative prior distribution $\Gamma (10^{-3},10^{-3})$, whereas for $\tau_{k}$, *k*=1,2,3,4, we assume $\Gamma (10^{-2},10^{-2})$. The priors for parameters $\alpha_{odt}^{k}$ are weakly informative normal densities, $N(0,10^{-2})$.

## 5 Results

The model was developed in the MATLAB software. The posterior characteristics were computed by using a Markov chain Monte Carlo sample of 300000 iterations, allowing for a burn‐in. We used a slice sampler (Neal, 2003) embedded in the Gibbs sampler (Geman and Geman, 1984) to draw samples from the posterior. The supplementary on‐line material contains details on the Markov chain Monte Carlo method that was used and MATLAB code, as well as auto‐correlation functions and cumulative mean plots of chains (Yu and Mykland, 1998) for selected model parameters.

### 5.1 Goodness of fit

**Figure 5:**
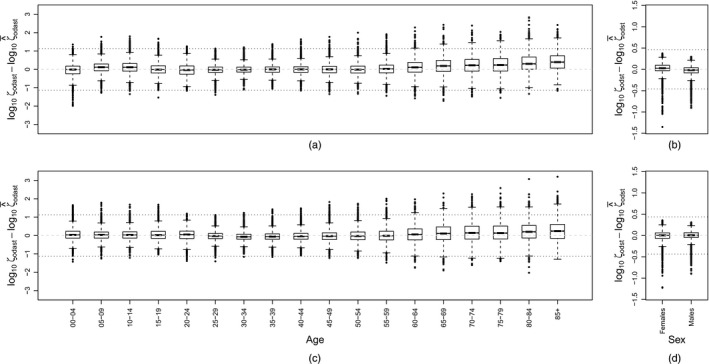
Distribution of differences in log‐transformed proportions for *ζ* and estimated means $\overset{¯}{\hat{\zeta }}$, by age and by sex (the dotted lines are the root‐mean‐squared errors of the differences (the data in (a) and (c) are not duplicated in (b) and (d)): (a), (b) emigration; (c), (d) immigration

The goodness of fit of the model to the data is assessed by generating samples from the predictive posterior distributions of the observed age–sex proportions, denoted by

$${\hat{\zeta }}_{odast}={\hat{z}}_{odast}/{\hat{z}}_{od++t},$$

and comparing common logarithms of their means, $\text{log}(\overset{¯}{\hat{\zeta }})$ with the common logarithms of the non‐zero data, i.e.  log (*ζ*). In Fig. 5, Tukey boxplots (McGill *et al*., 1978) of the differences $\text{log}(\zeta )-\text{log}(\overset{¯}{\hat{\zeta }})$ are presented for the available data on emigration and immigration with both age and sex (Figs 5(a) and 5(c)) and sex‐only (Figs 5(b) and 5(d)) profiles included. Two horizontal dotted lines are plotted at 3 standard deviations above and below zero, where the standard deviation is estimated by the root mean square of the sum of differences $\text{log}(\zeta )-\text{log}(\overset{¯}{\hat{\zeta }})$. A difference of 1 means that the proportion that is observed in the data is tenfold larger than the estimated mean. Ideally, the boxplots would be symmetric around zero, with outliers distributed uniformly for all age groups, and no visible differences between emigration and immigration.

For the age–sex data, which are presented in Figs 5(a) and 5(c), we observe a slight negative bias of the means for the older age groups, although zero lies within the box (an interquartile range) for all age groups but the oldest for emigration. The older age groups (ages 60 years and older) also have a higher proportion of differences greater than 3 standard deviations from zero, though, because this excess variability occurs in regions of the data where the counts are low, actual discrepancies in flows are negligible. There are no large discrepancies between emigration and immigration data. For the cases where only the sex profiles were available (Figs 5(b) and 5(d)), we observe that the differences are centred near zero with the negative outliers larger than the positive outliers, thus implying a slight overestimation by the model.

### 5.2 Country‐specific observations

**Figure 6:**
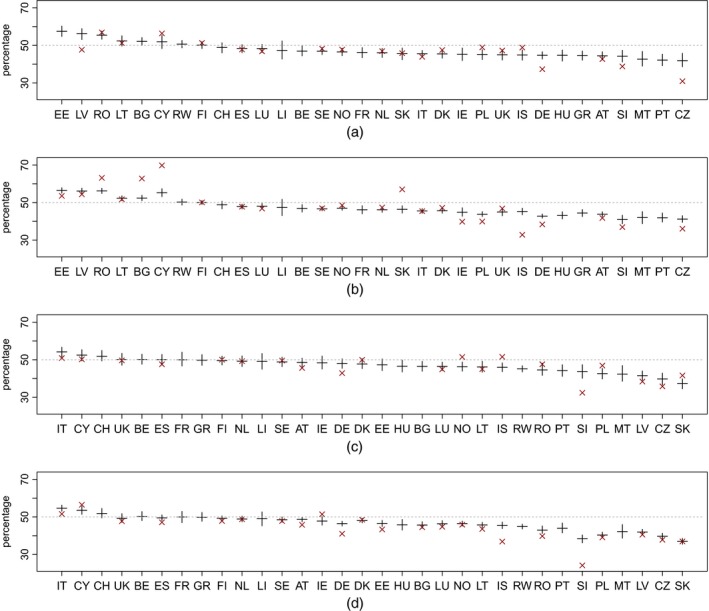
Mean percentages of emigration and immigration of females (horizontal) and 95% density interval (vertical) (×, reported data; countries on the *x*‐axis are ranked according to the mean female percentages estimated during the 2002–2003 period) (AT, Austria; BE, Belgium; BG, Bulgaria; CH, Switzerland; CY, Cyprus; CZ, Czech Republic; DE, Germany; DK, Denmark; EE, Estonia; ES, Spain; FI, Finland; FR, France; GR, Greece; HU, Hungary; IE, Republic of Ireland; IS, Iceland; IT, Italy; LI, Liechtenstein; LT, Lithuania; LU, Luxembourg; LV, Latvia; MT, Malta; NL, Netherlands; NO, Norway; PL, Poland; PT, Portugal; RO, Romania; SE, Sweden; SI, Slovenia; SK, Slovakia; UK, the UK; RW, rest of the world): (a) emmigration, 2002–2003; (b) emigration, 2004–2008; (c) immigration, 2002–2003; (d) immigration, 2004–2008

In Table 2 and Fig. 6, we present the mean percentages of emigration and immigration of females for each of the 31 countries in the EU and EFTA for the years before the 2004 EU enlargement (2002–2003) and afterwards (2004–2008). The largest discrepancies between the estimated mean and observed percentages are found with the 2004–2008 emigration flows. This can be partially explained by shifts in the proportions of migration of females (for example, see emigration from Latvia and Slovakia or immigration to Norway and Iceland).

**Table 2:** Mean percentages of female emigration and immigration in total for each country in the EU and EFTA, 2002–2003 and 2004–2008

*Country*	*Total emigration (%)*				*Total immigration (%)*			
	*Estimates*		*Data*		*Estimates*		*Data*	
	*2002–2003*	*2004–2008*	*2002–2003*	*2004–2008*	*2002–2003*	*2004–2008*	*2002–2003*	*2004–2008*
Austria	44	44	43	42	48	49	46	46
Belgium	47	47			50	50		
Bulgaria	52	52		63	47	46		45
Switzerland^a^	49	49			52	52		
Cyprus	52	55	56	70	52	54	50	56
Czech Republic	42	41	31	36	40	40	36	38
Germany	45	43	37	38	48	47	43	41
Denmark	45	46	48	47	48	48	50	49
Estonia	58	57		54	47	47		43
Spain	48	48	48	48	50	50	48	47
Finland	50	50	51	50	50	49	50	48
France	46	46			50	50		
Greece	44	44			50	50		
Hungary	45	43			47	46		
Republic of Ireland	45	45		40	48	48		51
Iceland^a^	45	45	49	33	46	46	52	37
Italy	46	46	44	45	54	55	51	52
Liechstenstein^a^	47	47			50	49		
Lithuania	52	52	51	52	46	46	45	44
Luxembourg	48	48	47	47	46	46	45	45
Latvia	56	56	48	54	41	42	38	41
Malta	42	42			42	42		
Netherlands	46	46	47	47	49	49	49	49
Norway^a^	46	47	48	49	46	47	51	46
Poland	45	44	49	40	43	40	47	39
Portugal	42	42			44	44		
Romania	56	56	57	63	45	43	48	40
Sweden	47	47	48	47	49	49	50	48
Slovenia	44	41	39	37	44	38	32	24
Slovakia	46	46	46	57	37	37	42	37
UK	45	45	47	47	50	49	50	48
Rest of the world	51	50			45	45		

a EFTA country.

The model estimates that males prevail in most of the flows for both emigration and immigration, although, for some countries, the results are inconclusive (i.e. the 95% density interval includes 50%). The largest estimated percentages of emigration of females are observed in Estonia, Latvia, Romania and Bulgaria, whereas, for males, in the Czech Republic, Portugal, Malta and Slovenia. Countries that attracted a large proportion of female migrants were Italy, Cyprus and Switzerland. Countries that were destinations for large shares of male migrants included Slovakia, Slovenia, Poland and the Czech Republic.

The differences in the sex distributions of emigration and immigration appear marginal but slightly higher percentages of females are observed in the immigration flows to western Europe, e.g. the UK and Germany. These results contrast with a generally higher percentage of women in the flows from central and eastern Europe (e.g. Romania, Estonia or Latvia).

**Figure 7:**
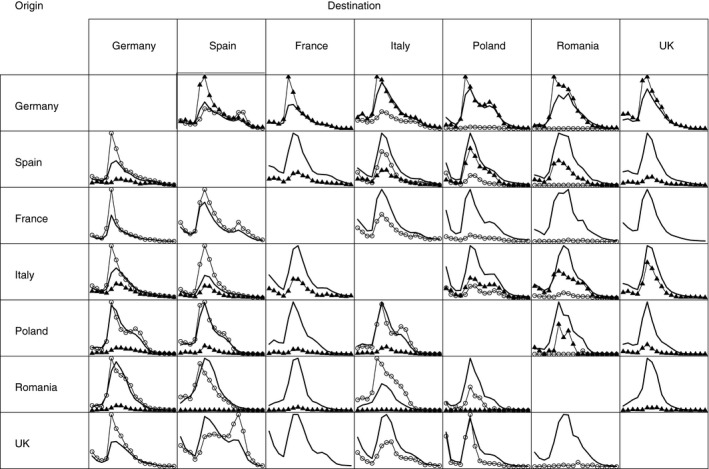
Medians of flows by age for females for the seven largest countries, 2008: estimates (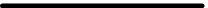), reported emigration (▵) and immigration data (◯)

In Fig. 7, median estimates of age‐specific migration among the seven largest EU and EFTA countries (i.e. Germany, Spain, France, the UK, Italy, Poland and Romania) are presented and compared with reported figures for the year 2008. Overall, we find that the estimated age patterns of migration coincide with our general expectations in terms of levels and age profiles. Most of the differences between the estimated and reported figures are found in the young adult age groups. Consider, for example, the flows from and to Germany, which is a country that provides good data but overstates the level of migration by not specifying a time criterion in its measurements. As expected, our harmonized median estimates are lower than the reported figures for Germany. As another example, consider the flows from and to Romania and Poland. Here, our median estimates are considerably higher than their reported figures, which is also expected since both countries impose a very restrictive ‘permanent’ definition of migration. For countries with no data or very weak data, the median estimates have age profiles and levels that appear plausible, e.g. a slight retirement peak in the migration from the UK to France or to Spain but none for the opposite flow.

**Figure 8:**
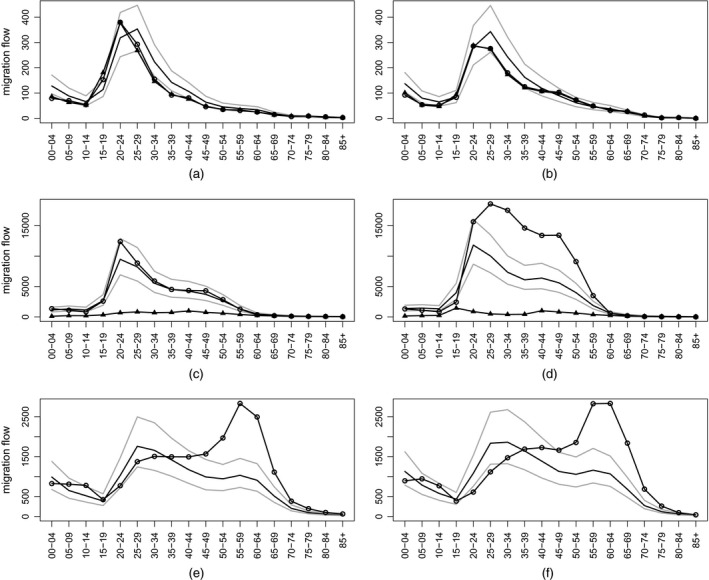
Age–sex profiles of reported migration from (a), (b) Finland to Sweden, (c), (d) Poland to Germany and (e), (f) the UK to Spain, 2006—posterior medians (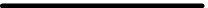), 80% predictive intervals (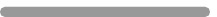), reports by sending country (▵) and reports by receiving country (◯): (a), (c), (e) females; (b), (d), (f) males

To illustrate some of the detailed age‐ and sex‐specific migration estimates, we have selected three flows representing high quality data (from Finland to Sweden), mixed quality data (from Poland to Germany) and single‐country reported data (from the UK to Spain). The median, first and ninth decile estimates by age and sex are presented in Fig. 8 for 2006. For the high quality data case, we observe that the estimated age profiles are similar to the observed data but with slightly higher values and a wider labour force peak. The higher levels are a consequence of the OD measurement model which included an expert‐based parameter for undercount. For the case with mixed quality data, our estimates lie mostly between the two reported figures, albeit closer to those provided by Germany. Lastly, for the case where only one report was available, our estimates have higher levels of migration of young adults and much lower levels of migration in the retirement age groups. The large hump in retirement ages reported by the Spanish register is not propagated in the results. This is a result of borrowing strength from the data on emigration to Spain reported by other countries, such as Germany (see Fig. 7), where the retirement migration was relatively low. The reported Spanish figures may reflect tourism and short duration retirement moves rather than migration *per se* (see, for example, Williams *et al*. (2000)).

## 6 Conclusions

We have presented a framework for estimating the distribution of the age and sex‐specific patterns of international migration flows. This framework has been applied to obtain flows between the 31 EU and EFTA countries from 2002 to 2008. The hierarchical Bayesian model combines age and sex profiles from both sending and receiving countries to provide a harmonized set of detailed migration flows with measures of uncertainty. We have also demonstrated a useful respecification of the multinomial model as a Poisson model with overdispersion.

The contributions of this paper are fourfold. First, we have developed an integrated model for distributing a matrix of international migration flows by age and sex based on incomplete and inconsistent information. This development extends the work by Raymer *et al*. (2013), which focused on measurement and spatial patterns of migration. Second, we have compared our estimates against reported values and identified where important differences arise. Third, we have shown how our results can be used to understand better the migration patterns in the EU and EFTA. The resulting estimates can also be used to improve current population estimation methods. Finally, we have provided a base for countries to improve their statistics on migration as required in the 2007 regulation on migration statistics that was passed by the European Parliament.

Our methodology is based on the notion of combining data across national statistical institutes, which allows countries to improve their migration statistics further. The estimates in this paper stop in the year 2008. Since then, the implementation of new EU regulations has altered how migration data are reported by national statistical institutes to Eurostat. Now, these agencies are required to provide migration flow statistics that are harmonized to a common definition. However, they are not obliged to change their data collection procedures and may use ‘scientifically based and well documented statistical estimation methods’ (article 9 of Regulation 862/2007 of the European Parliament and of the Council of July 11th, 2007) to augment their existing data. Although the new harmonization methods that have been adopted by national statistical institutes would probably not impact the estimation of the age–sex profiles of migration presented in this paper, they will affect the measurement model that is used to estimate the origin–destination flows in Raymer *et al*. (2013).

Future research in the field of migration estimation should focus on further refinements and expansions of the framework that is presented in this paper. First, this work should be continued and updated to cover a more recent period to identify whether the financial crisis has caused any subsequent changes to the migration patterns. Second, we included countries in the EU and EFTA. This could be expanded to incorporate other countries, world regions or even subnational areas (Dennett and Wilson, 2013). The model could be adapted to estimate domestic (within‐country) migration flows where data from censuses, administrative registers and surveys are combined (see for example Smith *et al*. (2010)). If reliable data become available, the model framework can be extended to include the specification of different types of migration, which would greatly enhance our understanding of the migration patterns motivated by, say, education, employment or family reunion (De Beer, 2008). Finally, the framework could be used to forecast future migration, whereby the time series of estimates provide a basis for extrapolation. The future estimates could then be included in population projections or for testing policy scenarios.

## Supporting information

‘Integrated modelling of age and sex patterns of European migration: Supplementary Online Material’.
