# Supplementary material for: Integrated modelling of age and sex patterns of European migration
Source: J R Stat Soc Ser A Stat Soc. 2016 Jan 22;179(4):1007–24. doi: 10.1111/rssa.12177 (PMC5053248; doi:10.1111/rssa.12177)
Supplement: Supplementary file 1 — ‘Integrated modelling of age and sex patterns of European migration: Supplementary Online Material’. [file RSSA-179-1007-s001.pdf]

Integrated modelling of age and sex patterns of European migration

Supplementary Online Material

Arkadiusz Wiśniowski*†[1] Jonathan J. Forster† Peter W.F. Smith†
Jakub Bijak† James Raymer‡

October 23, 2015

A Sampling from the posterior distribution

The model was developed in MATLAB software. Samples from the posterior distribution were drawn by using Markov Chain Monte Carlo (MCMC) with a slice sampler (Neal 2003) implemented for the Poisson part of the posterior and embedded in the Gibbs sampler (Geman and Geman 1984). The sample of 300,000 iterations with burn-in of 300,000 was used. The time required for computation was approximately 14-18 hours (depending on the other processes running simultaneously) on a Windows 7 machine with eight 3.4GHz CPUs and 16GB RAM.

B Mixing evidence

Autocorrelation functions (ACF) and histograms based on the MCMC sample after burn-in and cumulative mean (CM) plots (Yu and Mykland 1998) of entire chains for selected model parameters are presented in Figures 1 and 2.

C MATLAB code

```
% reading in the ODAST data table (18*32*31*2 x 7)
% contact authors for the files with the data
    z1 = csvread('Data_sources/Data_z1_ODAS.csv');
```

*School of Social Sciences, University of Manchester
†ESRC Centre for Population Change, University of Southampton
‡Australian Demographic and Social Research Institute, Australian National University
[1]Email: a.wisniowski@manchester.ac.uk

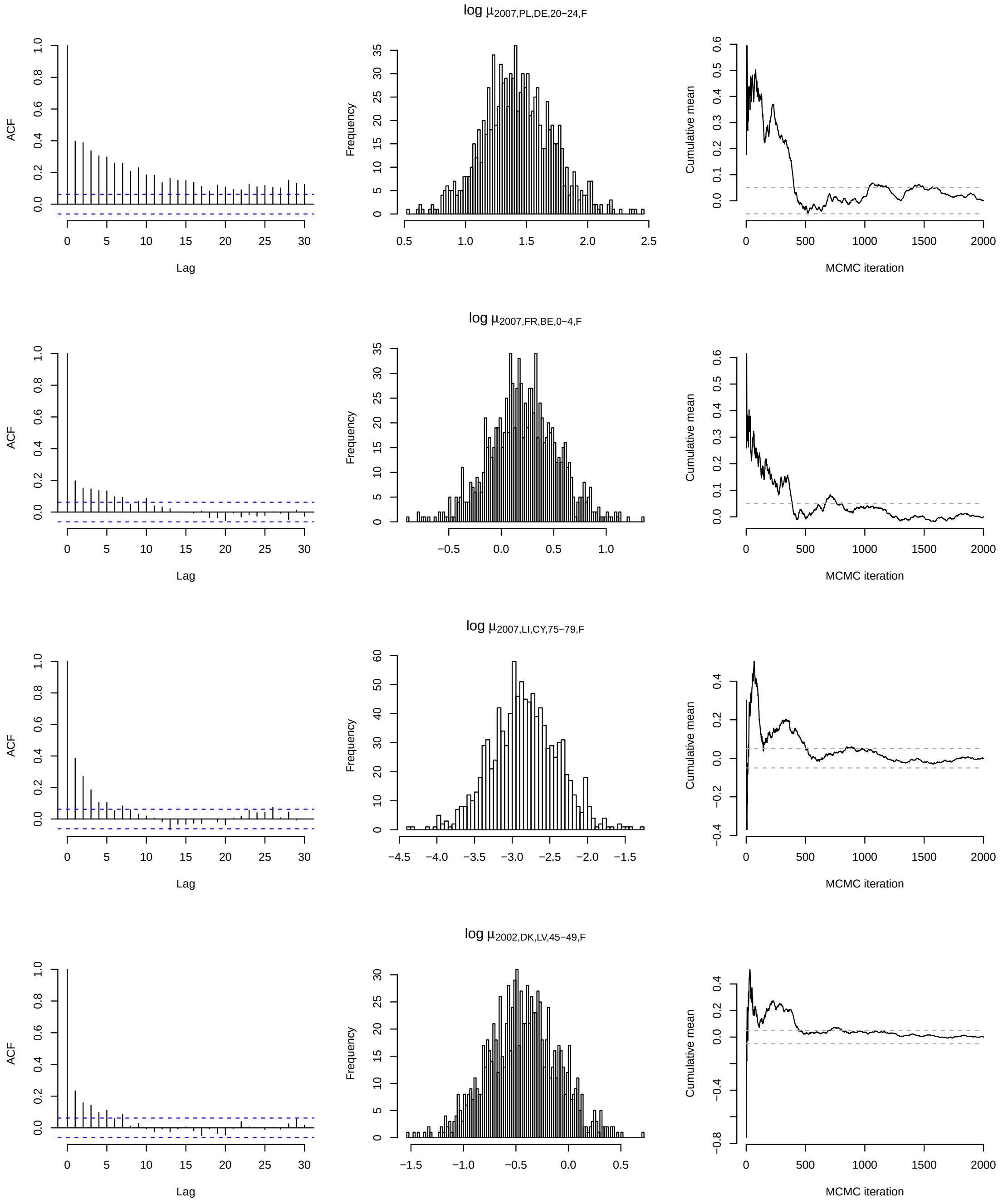

Figure 1: Autocorrelation functions (left), histograms (middle) and cumulative mean (right) plots for selected logarithms of the true flows. ACFs and histograms are based on the sample with burn-in removed.

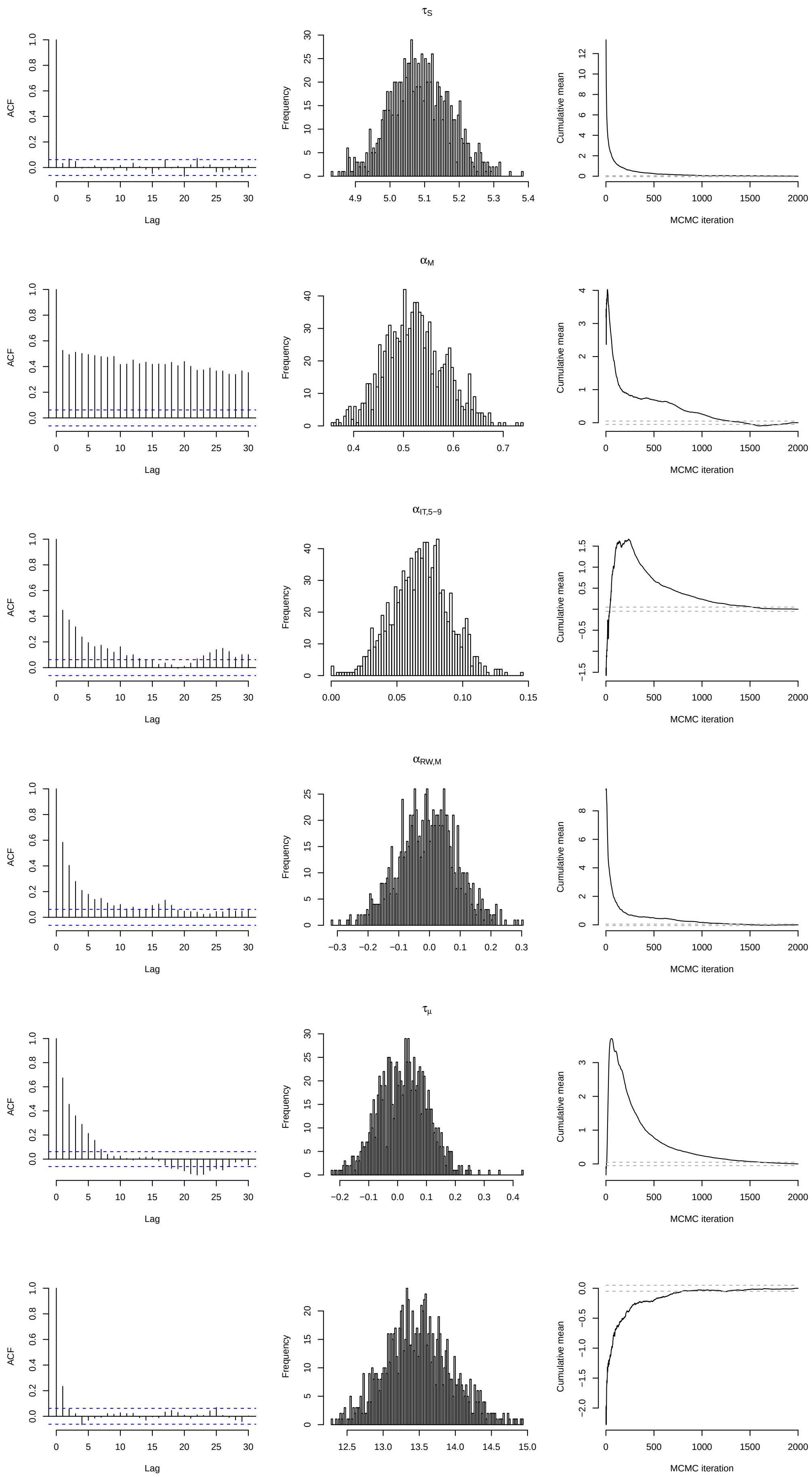

Figure 2: Autocorrelation functions (left), histograms (middle) and cumulative mean (right) plots for selected model parameters. ACFs and histograms are based on the sample with burn-in removed.

```
    z2 = csvread('Data_sources/Data_z2_ODAS.csv');
    zS=reshape(z1,18,32,31,2,7);
    zR=reshape(z2,18,31,32,2,7);
% 0.1 denotes missing observation
    zS(:,:,32,:,:) = 0.1;
    zR(:,32,:,:,:) = 0.1;
    for i = 1:32
        zS(:,i,i,:,:) = 0.1;
        zR(:,i,i,:,:) = 0.1;
    end
    % transform size to (TODAS) array
    zS=permute(zS,[5 3 2 1 4]);
    zR=permute(zR,[5 3 2 1 4]);

% reading in the OD.ST data (sex-only )
% contact authors for the files with the data
    z1s = csvread('Data_sources/Data_z1_ODSnew.csv');
    z2s = csvread('Data_sources/Data_z2_ODSnew.csv');
    zSs=reshape(z1s,32,31,2,7);
    zRs=reshape(z2s,31,32,2,7);
    zSs(:,32,:,:) = 0.1;
    zRs(32,:,:,:) = 0.1;
    for i = 1:32
        zSs(i,i,:,:) = 0.1;
        zRs(i,i,:,:) = 0.1;
    end
    % t o d a s
    zSs=permute(zSs,[4 2 1 3]);
    zRs=permute(zRs,[4 2 1 3]);
    zSs(zS(:,:,:,1,:)~=0.1)=0.1;
    zRs(zR(:,:,:,1,:)~=0.1)=0.1;

    %indices for sampling lvSs and lvRs
    indSs = find(zSs==0.1);
    indRs = find(zRs==0.1);
    indS1s = find(zSs~=0.1);
    indR1s = find(zRs~=0.1);
    lS1s=size(indS1s,1);
    lR1s=size(indR1s,1);

    zSsA=permute(repmat(zSs,[1,1,1,1,18]),[1 2 3 5 4]);
    zRsA=permute(repmat(zRs,[1,1,1,1,18]),[1 2 3 5 4]);

    %indices for sampling lmu
    i00 = find(zS==0.1 & zR==0.1 & zSsA==0.1 & zRsA==0.1);
    iS0 = find(zS~=0.1 & zR==0.1 & zSsA==0.1 & zRsA==0.1);
    i0R = find(zS==0.1 & zR~=0.1 & zSsA==0.1 & zRsA==0.1);
    iSR = find(zS~=0.1 & zR~=0.1 & zSsA==0.1 & zRsA==0.1);
    iS00R = find(zS~=0.1 & zR==0.1 & zSsA==0.1 & zRsA~=0.1);
    i0RS0 = find(zS==0.1 & zR~=0.1 & zSsA~=0.1 & zRsA==0.1);
    i00S0 = find(zS==0.1 & zR==0.1 & zSsA~=0.1 & zRsA==0.1);
```

```
    i000R = find(zS==0.1 & zR==0.1 & zSsA==0.1 & zRsA~=0.1);
    i00SR = find(zS==0.1 & zR==0.1 & zSsA~=0.1 & zRsA~=0.1);

    iS00Rs = find(squeeze(zS(:,:,:,1,:)~=0.1) & squeeze(zR(:,:,:,1,:)==0.1)...
        & zSs==0.1 & zRs~=0.1);
    i0RS0s = find(squeeze(zS(:,:,:,1,:)==0.1) & squeeze(zR(:,:,:,1,:)~=0.1)...
        & zSs~=0.1 & zRs==0.1);
    i00S0s = find(squeeze(zS(:,:,:,1,:)==0.1) & squeeze(zR(:,:,:,1,:)==0.1)...
        & zSs~=0.1 & zRs==0.1);
    i000Rs = find(squeeze(zS(:,:,:,1,:)==0.1) & squeeze(zR(:,:,:,1,:)==0.1)...
        & zSs==0.1 & zRs~=0.1);
    i00SRs = find(squeeze(zS(:,:,:,1,:)==0.1) & squeeze(zR(:,:,:,1,:)==0.1)...
        & zSs~=0.1 & zRs~=0.1);

    liS00R = size(iS00R,1);
    li0RS0 = size(i0RS0,1);
    li00S0 = size(i00S0,1);
    li000R = size(i000R,1);
    li00SR = size(i00SR,1);
    liS00Rs = size(iS00Rs,1);
    li0RS0s = size(i0RS0s,1);
    li00S0s = size(i00S0s,1);
    li000Rs = size(i000Rs,1);
    li00SRs = size(i00SRs,1);

    %indices for sampling lvS and lvR
    indS = find(zS==0.1);
    indR = find(zR==0.1);
    indS1 = find(zS~=0.1);
    indR1 = find(zR~=0.1);
    lS1=size(indS1,1);
    lR1=size(indR1,1);

    %indices for sampling a_odtK
    im1 = find(zS(:,:,:,1,1)~=0.1);
    im1s = find(zSs(:,:,:,1)~=0.1);
    im2 = find(zR(:,:,:,1,1)~=0.1);
    im2s = find(zRs(:,:,:,1)~=0.1);
%%%%%%%%%%%%%%%%%%%%%%%% END of DATA %%%%%%%%%%%%%%%%%%

%monitored variables
    tauA(1:9,1:2000)=NaN;
    aA1(1:18,1:2000)=NaN;
    aS1(1:2,1:2000)=NaN;
    lmuA(1:7,1:32,1:32,1:18,1:2,1:2000)=NaN;
    aodtk(1:7,1:32,1:32,1:2,1:2000)=NaN;
    a_daA(1:32,1:18,1:2000)=NaN;
    a_dsA(1:32,1:2,1:2000)=NaN;
    a_oaA(1:32,1:18,1:2000)=NaN;
    a_osA(1:32,1:2,1:2000)=NaN;

```

```
    % initial values
    T=7;
    r=31;
    vS(1:7,1:32,1:32,1:18,1:2)=zS/2+1;
    vR(1:7,1:32,1:32,1:18,1:2)=zR/2+1;
    lvS=log(vS);
    lvR=log(vR);
    lvS(indS)=NaN;
    lvR(indR)=NaN;
    vSs(1:7,1:32,1:32,1:2)=zSs/2+1;
    vRs(1:7,1:32,1:32,1:2)=zRs/2+1;
    lvSs=log(vSs);
    lvRs=log(vRs);
    lvSs(indSs)=NaN;
    lvRs(indRs)=NaN;
    lmu(1:7,1:32,1:32,1:18,1:2)=log((zS+zR+1)/2+1);
    tauS=1;
    tauR=1;
    tauSs=1;
    tauRs=1;
    tau_mu=1;

    m711(1:7,1:32,1:32,1:18,1:2) = NaN;
    m712(1:7,1:32,1:32,1:18,1:2) = NaN;
    m713(1:7,1:32,1:32,1:2) = NaN;
    m714(1:7,1:32,1:32,1:2) = NaN;
    m721(1:7,1:32,1:32) = NaN;
    m722(1:7,1:32,1:32) = NaN;

    a_odtS(1:7,1:32,1:32,1:18,1:2)=0;
    a_odtR(1:7,1:32,1:32,1:18,1:2)=0;
    a_odtS1(1:7,1:32,1:32)=NaN;
    a_odtR1(1:7,1:32,1:32)=NaN;
    a_da(1:7,1:32,1:32,1:18,1:2)=0;
    a_oa(1:7,1:32,1:32,1:18,1:2)=0;
    a_ds(1:7,1:32,1:32,1:18,1:2)=0;
    a_os(1:7,1:32,1:32,1:18,1:2)=0;
    a_a(1:18)=0;
    a_s(1:2)=0;
    tau1=1;    %DA
    tau2=1;    %DS
    tau3=1;    %OA
    tau4=1;    %OS


% Prior hyperparameters
    a31 = 9*32+0.01;
    a32 = 32+0.01;
    ta = 0.01;
    ts = 0.01;
    a1 = 18*31*32*7 + 0.001;
```

```
    a2 = lS1/2+ 0.001;
    a3 = lR1/2+ 0.001;
    a4 = size(indS1s,1)/2 + 0.001;
    a5 = size(indR1s,1)/2 + 0.001;

% Gibbs sampler
     for i = 1:600000

    % slice sampling v (Poisson-log-normal ODAST)
        w1S=zS(indS1).*lvS(indS1)-exprnd(1,lS1,1);
        w2S=-exp(lvS(indS1))-exprnd(1,lS1,1);
        w3S=-tauS*0.5*(lvS(indS1)-lmu(indS1)-a_odtS(indS1)).^2-...
            exprnd(1,lS1,1);
        lvS(indS1) = unifrnd(max(w1S./zS(indS1),...
            a_odtS(indS1)+lmu(indS1)-sqrt(-2*w3S/tauS)),...
            min(log(-w2S), a_odtS(indS1)+lmu(indS1)+sqrt(-2*w3S/tauS)),...
            lS1,1 );

        w1R=zR(indR1).*lvR(indR1)-exprnd(1,lR1,1);
        w2R=-exp(lvR(indR1))-exprnd(1,lR1,1);
        w3R=-tauR*0.5*(lvR(indR1)-lmu(indR1)-a_odtR(indR1)).^2-...
            exprnd(1,lR1,1);
        lvR(indR1) = unifrnd( max(w1R./zR(indR1), a_odtR(indR1)+lmu(indR1)...
            -sqrt(-2*w3R/tauR)),...
            min(log(-w2R), a_odtR(indR1)+lmu(indR1)+sqrt(-2*w3R/tauR)),...
            lR1,1);

     % slice sampling vs (Poisson-log-normal OD.ST)
        w1Ss=zSs(indS1s).*lvSs(indS1s)-exprnd(1,lS1s,1);
        w2Ss=-exp(lvSs(indS1s))-exprnd(1,lS1s,1);
        maxlmu=max(lmu,[],4);
        sp1=squeeze(maxlmu+log(sum(exp(lmu-...
            repmat(maxlmu,[1,1,1,18,1])),4)));
        a_odtS2=squeeze(a_odtS(:,:,:,1,:));
        w3Ss=-tauSs*0.5*(lvSs(indS1s)-sp1(indS1s)-a_odtS2(indS1s)).^2-...
            exprnd(1,lS1s,1);
        lvSs(indS1s) = unifrnd( max(w1Ss./zSs(indS1s),...
            a_odtS2(indS1s)+sp1(indS1s)-sqrt(-2*w3Ss/tauSs)),...
            min(log(-w2Ss), a_odtS2(indS1s)+sp1(indS1s)+sqrt(-2*w3Ss/tauSs)),...
            lS1s,1 );

        w1Rs=zRs(indR1s).*lvRs(indR1s)-exprnd(1,lR1s,1);
        w2Rs=-exp(lvRs(indR1s))-exprnd(1,lR1s,1);
        a_odtR2=squeeze(a_odtR(:,:,:,1,:));
        w3Rs=-tauRs*0.5*(lvRs(indR1s)-sp1(indR1s)-a_odtR2(indR1s)).^2-...
            exprnd(1,lR1s,1);
        lvRs(indR1s) = unifrnd( max(w1Rs./zRs(indR1s), ...
            a_odtR2(indR1s)+sp1(indR1s)-sqrt(-2*w3Rs/tauRs)),...
            min(log(-w2Rs), a_odtR2(indR1s)+sp1(indR1s)+...
            sqrt(-2*w3Rs/tauRs)),lR1s,1);

```

```
% conditional for mu_odast
    m00 = (tau_mu*(a_da(i00) + a_ds(i00) + a_oa(i00) + a_os(i00)))./...
        tau_mu;
    sd00 = 1/sqrt(tau_mu); %sd

    mS0 = (tauS*(lvS(iS0)-a_odtS(iS0)) +...
        tau_mu*(a_da(iS0) + a_ds(iS0) + a_oa(iS0) + a_os(iS0)))./...
        (tauS +tau_mu);
    sdS0 = 1/sqrt(tauS + tau_mu); %sd

    m0R = (tauR*(lvR(i0R)-a_odtR(i0R)) +...
        tau_mu*(a_da(i0R) + a_ds(i0R) + a_oa(i0R) + a_os(i0R)))./...
        (tauR +tau_mu);
    sd0R = 1/sqrt(tauR + tau_mu); %sd

    mSR = (tauR*(lvR(iSR)-a_odtR(iSR)) + tauS*(lvS(iSR)-a_odtS(iSR)) +...
        tau_mu*(a_da(iSR) + a_ds(iSR) + a_oa(iSR) + a_os(iSR)))./...
        (tauS + tauR + tau_mu);
    sdSR = 1/sqrt(tauS + tauR + tau_mu); %sd

    lvSs1=permute(repmat(lvSs,[1,1,1,1,18]),[1 2 3 5 4]);
    lvRs1=permute(repmat(lvRs,[1,1,1,1,18]),[1 2 3 5 4]);
    sp2=repmat(sum(exp(lmu),4),[1,1,1,18,1])-exp(lmu);
    spf=log(repmat(sum(exp(lmu),4),[1,1,1,18,1]));
    spf1=log(sum(exp(lmu),4));

    %S00R
    w1 = -0.5*tauS*(-lvS(iS00R)+a_odtS(iS00R)+lmu(iS00R)).^2-...
        exprnd(1,liS00R,1);
    w2 = -0.5*tau_mu*(lmu(iS00R) - a_da(iS00R) - a_ds(iS00R) -...
        a_oa(iS00R) - a_os(iS00R)).^2-exprnd(1,liS00R,1);

    a_odtRh = squeeze(a_odtR(:,:,:,1,:));
    w3a = -0.5*tauRs*(lvRs(iS00Rs)-a_odtRh(iS00Rs)-spf1(iS00Rs)).^2-...
        exprnd(1,liS00Rs,1);
    w3=[kron(ones(18,1),w3a(1:(liS00Rs/2))); ...
        kron(ones(18,1),w3a((liS00Rs/2+1):end))];

    lmu(iS00R) = unifrnd(max(-sqrt(-2*w1/tauS)+lvS(iS00R)-a_odtS(iS00R),...
        max(a_da(iS00R)+ a_ds(iS00R)+ a_oa(iS00R)+ a_os(iS00R)-...
        sqrt(-2*w2/tau_mu), log((exp(-sqrt(-2*w3/tauRs)+...
        lvRs1(iS00R)-a_odtR(iS00R))-sp2(iS00R)).*...
        ((exp(-sqrt(-2*w3/tauRs)+lvRs1(iS00R)-a_odtR(iS00R))-...
        sp2(iS00R))>0)))),...
        min(sqrt(-2*w1/tauS)+lvS(iS00R)-a_odtS(iS00R),min(...
        a_da(iS00R)+ a_ds(iS00R)+ a_oa(iS00R)+ a_os(iS00R)+ ...
        sqrt(-2*w2/tau_mu),log(exp(sqrt(-2*w3/tauRs)+lvRs1(iS00R)-...
        a_odtR(iS00R))-sp2(iS00R)))), liS00R,1);

    %0RS0
```

```
    w1 = -0.5*tauR*(-lvR(i0RS0)+a_odtR(i0RS0)+lmu(i0RS0)).^2-exprnd(1,li0RS0,1);
    w2 = -0.5*tau_mu*(lmu(i0RS0) - a_da(i0RS0) - a_ds(i0RS0) - a_oa(i0RS0)...
        - a_os(i0RS0)).^2-exprnd(1,li0RS0,1);
    w3 = -0.5*tauSs*(lvSs1(i0RS0)-a_odtS(i0RS0)-spf(i0RS0)).^2-exprnd(1,li0RS0,1);
    lmu(i0RS0) = unifrnd(max(-sqrt(-2*w1/tauR)+lvR(i0RS0)-a_odtR(i0RS0),max(...
        a_da(i0RS0)+ a_ds(i0RS0)+ a_oa(i0RS0)+ a_os(i0RS0)- sqrt(-2*w2/tau_mu),...
        log((exp(-sqrt(-2*w3/tauSs)+lvSs1(i0RS0)-a_odtS(i0RS0))-sp2(i0RS0))...
        .*((exp(-sqrt(-2*w3/tauSs)+lvSs1(i0RS0)-a_odtS(i0RS0))-sp2(i0RS0))>0)))),...
        min(sqrt(-2*w1/tauR)+lvR(i0RS0)-a_odtR(i0RS0),min(...
        a_da(i0RS0)+ a_ds(i0RS0)+ a_oa(i0RS0)+ a_os(i0RS0)+ sqrt(-2*w2/tau_mu),...
        log(exp(sqrt(-2*w3/tauSs)+lvSs1(i0RS0)-a_odtS(i0RS0))-sp2(i0RS0)))),...
        li0RS0,1);

    %00S0
    w2 = -0.5*tau_mu*(lmu(i00S0) - a_da(i00S0) - a_ds(i00S0) - a_oa(i00S0)...
        - a_os(i00S0)).^2-exprnd(1,li00S0,1);
    w3 = -0.5*tauSs*(lvSs1(i00S0)-a_odtS(i00S0)-spf(i00S0)).^2-exprnd(1,li00S0,1);
    lmu(i00S0) = unifrnd(...
        max(a_da(i00S0)+ a_ds(i00S0)+ a_oa(i00S0)+ a_os(i00S0)-...
        sqrt(-2*w2/tau_mu), log((exp(-sqrt(-2*w3/tauSs)+lvSs1(i00S0)...
        -a_odtS(i00S0))-sp2(i00S0)).*((exp(-sqrt(-2*w3/tauSs)+...
        lvSs1(i00S0)-a_odtS(i00S0))-sp2(i00S0))>0))),...
        min(a_da(i00S0)+ a_ds(i00S0)+ a_oa(i00S0)+ a_os(i00S0)+...
        sqrt(-2*w2/tau_mu), log(exp(sqrt(-2*w3/tauSs)+lvSs1(i00S0)-...
        a_odtS(i00S0))-sp2(i00S0))),li00S0,1);

    %000R
    w2 = -0.5*tau_mu*(lmu(i000R) - a_da(i000R) - a_ds(i000R) - a_oa(i000R)...
        - a_os(i000R)).^2-exprnd(1,li000R,1);
    w3 = -0.5*tauRs*(lvRs1(i000R)-a_odtR(i000R)-spf(i000R)).^2-exprnd(1,li000R,1);
    lmu(i000R) = unifrnd(...
        max(a_da(i000R)+ a_ds(i000R)+ a_oa(i000R)+ a_os(i000R)-...
        sqrt(-2*w2/tau_mu),...
        log((exp(-sqrt(-2*w3/tauRs)+lvRs1(i000R)-a_odtR(i000R))-sp2(i000R))...
        .*((exp(-sqrt(-2*w3/tauRs)+lvRs1(i000R)-a_odtR(i000R))-sp2(i000R))>0))),...
        min(a_da(i000R)+ a_ds(i000R)+ a_oa(i000R)+ a_os(i000R)+...
        sqrt(-2*w2/tau_mu),...
        log(exp(sqrt(-2*w3/tauRs)+lvRs1(i000R)-a_odtR(i000R))-sp2(i000R))),...
        li000R,1);

    %00SR
    w1 = -0.5*tauRs*(lvRs1(i00SR)-a_odtR(i00SR)-spf(i00SR)).^2-exprnd(1,li00SR,1);
    w2 = -0.5*tau_mu*(lmu(i00SR) - a_da(i00SR) - a_ds(i00SR) - a_oa(i00SR)...
        - a_os(i00SR)).^2-exprnd(1,li00SR,1);
    w3 = -0.5*tauSs*(lvSs1(i00SR)-a_odtS(i00SR)-spf(i00SR)).^2-exprnd(1,li00SR,1);
    lmu(i00SR) = unifrnd(...
        max(log((exp(-sqrt(-2*w1/tauRs)+lvRs1(i00SR)-a_odtR(i00SR))-sp2(i00SR))...
        .*((exp(-sqrt(-2*w1/tauRs)+lvRs1(i00SR)-a_odtR(i00SR))-...
        sp2(i00SR))>0)),max(a_da(i00SR)+ a_ds(i00SR)+ a_oa(i00SR)+ a_os(i00SR)- ...
        sqrt(-2*w2/tau_mu), log((exp(-sqrt(-2*w3/tauSs)+lvSs1(i00SR)-...
        a_odtS(i00SR))-sp2(i00SR))...
```

```
            .*((exp(-sqrt(-2*w3/tauSs)+lvSs1(i00SR)-a_odtS(i00SR))-sp2(i00SR))>0)))),...
            min(log(exp(sqrt(-2*w1/tauRs)+lvRs1(i00SR)-a_odtR(i00SR))-sp2(i00SR)),...
            min(a_da(i00SR)+ a_ds(i00SR)+ a_oa(i00SR)+ a_os(i00SR)+...
            sqrt(-2*w2/tau_mu),log(exp(sqrt(-2*w3/tauSs)+lvSs1(i00SR)-...
            a_odtS(i00SR))-sp2(i00SR)))), li00SR,1);

        % sampling log mu
        lmu(i00) = m00 + sd00*normrnd(0,1,size(i00,1),1);
        lmu(iS0) = mS0 + sdS0*normrnd(0,1,size(iS0,1),1);
        lmu(i0R) = m0R + sd0R*normrnd(0,1,size(i0R,1),1);
        lmu(iSR) = mSR + sdSR*normrnd(0,1,size(iSR,1),1);

            for k=1:32
                lmu(:,k,k,:,:) = NaN;
            end;
        sp1=squeeze(log(sum(exp(lmu),4)));

    % conditional for a_odtk
        %   t    o    d    a    s
        m711(indS1) = lvS(indS1) - lmu(indS1);
        m713(indS1s) = lvSs(indS1s) - sp1(indS1s);
        mhs11=sum(sum(m711,4),5);
        mhs12=sum(m713,4);
        m721(im1) = (tauS*mhs11(im1))./(tauS*2*18);  %mean
        m721(im1s) = (tauSs*mhs12(im1s))./(tauSs*2); %mean

        m712(indR1) = lvR(indR1) - lmu(indR1);
        m714(indR1s) = lvRs(indR1s) - sp1(indR1s);
        mhs21=sum(sum(m712,4),5);
        mhs22=sum(m714,4);
        m722(im2) = (tauR*mhs21(im2))./(tauR*2*18); %mean
        m722(im2s) = (tauRs*mhs22(im2s))./(tauRs*2); %mean

        % sampling
        a_odtS1(im1) = m721(im1) + 1/sqrt(tauS*2*18) * normrnd(0,1,size(im1));
        a_odtS1(im1s) = m721(im1s) + 1/sqrt(tauSs*2) * normrnd(0,1,size(im1s));
        a_odtR1(im2) = m722(im2) + 1/sqrt(tauR*2*18) * normrnd(0,1,size(im2));
        a_odtR1(im2s) = m722(im2s) + 1/sqrt(tauRs*2) * normrnd(0,1,size(im2s));
        a_odtS = repmat(a_odtS1,[1,1,1,18,2]);
        a_odtR = repmat(a_odtR1,[1,1,1,18,2]);

    % conditional for a_da
        %   t    o    d    a    s
        m11(1:7,1:32,1:32,1:18,1:2) = lmu - (a_ds + a_oa + a_os);
        sm1(1:32,1:18)=nansum(nansum(nansum(m11,1),2),5);
        m12(1:32,1:18) = (tau1*kron(a_a,ones(32,1))+tau_mu*sm1)./...
            (tau_mu*7*2*31+tau1); %mean
        sd1 = 1/sqrt(tau_mu*7*2*31+tau1); %sd
        % sampling
        a_da1 = m12 + sd1 * normrnd(0,1,32,18);
        a_da = permute(repmat(a_da1,[1,1,7,32,2]),[3,4,1,2,5]);
```

```
    % conditional for a_ds
        %   t    o   d    a    s
        m21(1:7,1:32,1:32,1:18,1:2) = lmu - (a_da + a_oa + a_os);
        sm2(1:32,1:2)=nansum(nansum(nansum(m21,1),2),4);
        m22(1:32,1:2) = (tau2*kron(a_s,ones(32,1))+tau_mu*sm2)./...
            (tau_mu*7*18*31+tau2); %mean
        sd2 = 1/sqrt(tau_mu*7*18*31+tau2); %sd
        % sampling
        a_ds1 = m22 + sd2 * normrnd(0,1,32,2);
        a_ds = permute(repmat(a_ds1,[1,1,7,32,18]),[3,4,1,5,2]);

    % conditional for a_oa
        %   t    o   d    a    s
        m31(1:7,1:32,1:32,1:18,1:2) = lmu - (a_ds + a_da + a_os);
        sm3(1:32,1:18)=nansum(nansum(nansum(m31,1),3),5);
        m32(1:32,1:18) = (tau3*kron(a_a,ones(32,1))+tau_mu*sm3)./...
            (tau_mu*7*2*31+tau3); %mean
        sd3 = 1/sqrt(tau_mu*7*2*31+tau3); %sd
        % sampling
        a_oa1 = m32 + sd3 * normrnd(0,1,32,18);
        a_oa = permute(repmat(a_oa1,[1,1,7,32,2]),[3,1,4,2,5]);

    % conditional for a_os
        %   t    o   d    a    s
        m41(1:7,1:32,1:32,1:18,1:2) = lmu - (a_da + a_oa + a_ds);
        sm4(1:32,1:2)=nansum(nansum(nansum(m41,1),3),4);
        m42(1:32,1:2) = (tau4*kron(a_s,ones(32,1))+tau_mu*sm4)./...
            (tau_mu*7*18*31+tau4); %mean
        sd4 = 1/sqrt(tau_mu*7*18*31+tau4); %sd
        % sampling
        a_os1 = m42 + sd4 * normrnd(0,1,32,2);
        a_os = permute(repmat(a_os1,[1,1,7,32,18]),[3,1,4,5,2]);

    % conditional for a_a
        m5(1:18) = (tau1*sum(a_da(1,1,:,:,1),3) + ...
            tau3*sum(a_oa(1,:,1,:,1),2))./...
            (ta+32*(tau1+tau3));    %mean
        sd5 = 1/sqrt(ta+32*(tau1+tau3)); %sd
        % sampling
        a_a(1) = 0;
        a_a(2:18) = m5(2:18) + sd5 * normrnd(0,1,1,17);

    % conditional for a_s
        m6(1:2) = (tau2*sum(a_ds(1,1,:,1,:),3) +...
            tau4*sum(a_os(1,:,1,1,:),2))./...
            (ts+32*(tau2+tau4));    %mean
        sd6 = 1/sqrt(ts+32*(tau2+tau4)); %sd
        % sampling
        a_s(1) = 0;
        a_s(2) = m6(2) + sd6 * normrnd(0,1,1);
```

```
    % conditional for tauS and tauR

        b21 = 1/(0.001+0.5*sum((lvS(indS1)-lmu(indS1)-a_odtS(indS1)).^2));
        b22 = 1/(0.001+0.5*sum((lvR(indR1)-lmu(indR1)-a_odtR(indR1)).^2));
        tauS = gamrnd(a2,b21,1);
        tauR = gamrnd(a3,b22,1);

    % conditional for tauSs and tauRs

        a_odtS2=squeeze(a_odtS(:,:,:,1,:));
        a_odtR2=squeeze(a_odtR(:,:,:,1,:));
        b21s = 1/(0.001+0.5*sum((lvSs(indS1s)-sp1(indS1s)-a_odtS2(indS1s)).^2));
        b22s = 1/(0.001+0.5*sum((lvRs(indR1s)-sp1(indR1s)-a_odtR2(indR1s)).^2));
        tauSs = gamrnd(a4,b21s,1);
        tauRs = gamrnd(a5,b22s,1);

    % conditionals for tau1, tau2, tau3, tau4
        %DA tau1    DS tau2  OA tau3   OS tau4
        b31 = 1/(0.01+0.5*nansum(nansum((squeeze(a_da(1,1,:,:,1)) -...
            kron(a_a,ones(32,1))).^2,1),2));
        b32 = 1/(0.01+0.5*nansum(nansum((squeeze(a_ds(1,1,:,1,:)) -...
            kron(a_s,ones(32,1))).^2,1),2));
        b33 = 1/(0.01+0.5*nansum(nansum((squeeze(a_oa(1,:,1,:,1)) -...
            kron(a_a,ones(32,1))).^2,1),2));
        b34 = 1/(0.01+0.5*nansum(nansum((squeeze(a_os(1,:,1,1,:)) -...
            kron(a_s,ones(32,1))).^2,1),2));
        tau1 = gamrnd(a31,b31,1);
        tau2 = gamrnd(a32,b32,1);
        tau3 = gamrnd(a31,b33,1);
        tau4 = gamrnd(a32,b34,1);


    % conditional for tau_mu
        b1(1) = 1/(0.001+0.5*nansum(...
            ((lmu(:) - a_da(:) - a_ds(:) - a_oa(:) - a_os(:)).^2)));
        tau_mu = gamrnd(a1,b1,1);

    % thinning - saving every 300th iteration
in=ceil(i/300);
        tauA(1:9,in)=[tau_mu, tauS,tauR,tauSs,tauRs,tau1,tau2,tau3,tau4]';
        aA1(1:18,in)=a_a';
        aS1(1:2,in)=a_s';
        aodtk(1:7,1:32,1:32,1,in)=a_odtS1;
        aodtk(1:7,1:32,1:32,2,in)=a_odtR1;
        a_daA(1:32,1:18,in)=a_da1;
        a_dsA(1:32,1:2,in)=a_ds1;
        a_oaA(1:32,1:18,in)=a_oa1;
        a_osA(1:32,1:2,in)=a_os1;
        lmuA(:,:,:,:,:,in)=lmu;
    % monitoring progress
```

```
    % if mod(i,300) == 0 i
    % end
     end
%%%%%%%%%%%%% END OF SAMPLING %%%%%%%%%%%


     % reading in OD model results (requires function bugs2mat
     % contact authors for the files with the data
S=bugs2mat('11_6871FINCODAindex.txt','11_6871FINCODAchain1.txt');
y1=permute(repmat(S.y(2:1001,:,:,:),[1,1,1,1,18,2]),[4,2,3,5,6,1]);
s1=repmat(sum(sum(exp(lmuA),4),5),[1,1,1,18,2,1]);
% producing flows by ODAST, counts (1000 iterations)
TY(1:7,1:32,1:32,1:18,1:2,1:1000)=exp(lmuA)./s1.*y1;
% producing flows by ODAST, rates (1000 iterations)
F(1:7,1:32,1:32,1:18,1:2,1:1000)=exp(lmuA)./s1;

% model validation and goodness of fit
% samples from predictve posterior distributions
% job carried out on cluster computer iridis 3
a_daS(1:32,1:18,1:1000)=permute(repmat(aA1(:,1001:2000),[1,1,32]),[3 1 2])...
    +permute(repmat(1./sqrt(tauA(6,1001:2000)),[32 1 18]),[1 3 2]).*...
    normrnd(0,1,32,18,1000);
a_dsS(1:32,1:2,1:1000)=permute(repmat(aS1(:,1001:2000),[1,1,32]),[3 1 2])...
    +permute(repmat(1./sqrt(tauA(7,1001:2000)),[32 1 2]),[1 3 2]).*...
    normrnd(0,1,32,2,1000);
a_oaS(1:32,1:18,1:1000)=permute(repmat(aA1(:,1001:2000),[1,1,32]),[3 1 2])...
    +permute(repmat(1./sqrt(tauA(8,1001:2000)),[32 1 18]),[1 3 2]).*...
    normrnd(0,1,32,18,1000);
a_osS(1:32,1:2,1:1000)=permute(repmat(aS1(:,1001:2000),[1,1,32]),[3 1 2])...
    +permute(repmat(1./sqrt(tauA(9,1001:2000)),[32 1 2]),[1 3 2]).*...
    normrnd(0,1,32,2,1000);
lmuAS(1:7,1:32,1:32,1:18,1:2,1:1000)=...
    permute(repmat(a_daS,[1,1,1,7,32,2]),[4,5,1,2,6,3])+...
    permute(repmat(a_dsS,[1,1,1,7,32,18]),[4,5,1,6,2,3])+...
    permute(repmat(a_oaS,[1,1,1,7,32,2]),[4,1,5,2,6,3])+...
    permute(repmat(a_osS,[1,1,1,7,32,18]),[4,1,5,6,2,3])+...
    permute(repmat(1./sqrt(tauA(1,1001:2000)'),[1,7,32,32,18,2]),...
    [2 3 4 5 6 1]).*normrnd(0,1,7,32,32,18,2,1000);
lvSS=lmuAS+permute(repmat(aodtk(:,:,:,1,1001:2000),[1 1 1 18 1 2]),...
    [1 2 3 4 6 5])+permute(repmat(1./sqrt(tauA(2,1001:2000)'),...
    [1,7,32,32,18,2]),[2 3 4 5 6 1]).*normrnd(0,1,7,32,32,18,2,1000);
lvRS=lmuAS+permute(repmat(aodtk(:,:,:,2,1001:2000),[1 1 1 18 1 2]),...
    [1 2 3 4 6 5])+permute(repmat(1./sqrt(tauA(3,1001:2000)'),...
    [1,7,32,32,18,2]),[2 3 4 5 6 1]).*normrnd(0,1,7,32,32,18,2,1000);
zsS=poissrnd(exp(lvSS));
zsR=poissrnd(exp(lvRS));
```
